# Magnetic hydrophobic‐charge induction adsorbents for the recovery of immunoglobulins from antiserum feedstocks by high‐gradient magnetic fishing

**DOI:** 10.1002/jctb.5599

**Published:** 2018-03-30

**Authors:** Cláudia SG Gomes, Adedayo Fashina, Alfred Fernández‐Castané, Timothy W Overton, Timothy J Hobley, Eirini Theodosiou, Owen RT Thomas

**Affiliations:** ^1^ Institute of Biotechnology and Biomedicine Technical University of Denmark Lyngby Denmark; ^2^ School of Chemical Engineering University of Birmingham Birmingham UK; ^3^ National Food Institute Technical University of Denmark Lyngby Denmark; ^4^ Aston Institute of Materials Research Aston University Birmingham UK

**Keywords:** direct capture, polyclonal and monoclonal antibodies, 4‐mercaptoethylpyridine, serum and plasma, complex unclarified bioprocess liquors

## Abstract

**BACKGROUND:**

The extraction of biopharmaceuticals from plasma and serum often employs overly complicated antiquated procedures that can inflict serious damage on especially prone protein targets and which afford low purification power and overall yields. This paper describes systematic development of a high‐gradient magnetic fishing process for recovery of immunoglobulins from unclarified antiserum.

**RESULTS:**

Non‐porous superparamagnetic particles were transformed into hydrophobic‐charge induction adsorbents and then used to recover immunoglobulins from rabbit antiserum feedstocks. Comprehensive characterisation tests conducted with variously diluted clarified antiserum on a magnetic rack revealed that immunoglobulin binding was rapid (equilibrium reached in <45 s), strong (K_d_ < 0.1 mg mL^‐1^), of high capacity (Q_max_ = 214 mg g^‐1^), and pH and ionic strength dependent. In a high‐gradient magnetic fishing process conducted with the same adsorbent, and a conventional ‘magnetic filter + recycle loop’ arrangement, >72% of the immunoglobulin present in an unclarified antiserum feed was recovered in 0.5 h in >3‐fold purified form.

**CONCLUSIONS:**

Fast magnetic particle based capture of antibodies from an unclarified high‐titre feed has been demonstrated. Efficient product recovery from ultra‐high titre bioprocess liquors by high‐gradient magnetic fishing requires that improved magnetic adsorbents displaying high selectivity, ultra‐high capacity and operational robustness are used with ‘state‐of‐the‐art’ rotor–stator magnetic separators. © 2018 The Authors. *Journal of Chemical Technology & Biotechnology* published by John Wiley & Sons Ltd on behalf of Society of Chemical Industry.

## INTRODUCTION

The markets for blood‐derived protein products are large, and growing, albeit not fast enough to meet escalating global needs.^1^, ^2^ Among recognised factors hindering growth are: increased competition from recombinant alternatives; high cost and limited procurement of pooled plasma starting material, meaning that several therapeutic products must be extracted from the same pool; and continued reliance on antiquated overly expensive and complex production processes.^2^, ^3^

Outdated purification methods also persist within the immunodiagnostics sector and in large‐scale commercial processing of animal plasmas. For example, multiple steps of ammonium sulphate precipitation, dialysis and ion exchange batch adsorption are commonly employed in the production of polyclonal antibodies from serum for diagnostic kit applications,^4^, ^5^, ^6^ and harsh precipitation principles are likewise common in recovery operations for target proteins from equine plasmas, often compromising the extraction of other protein products.^7^, ^8^, ^9^

Immunoglobulins are currently the most important blood‐derived product, accounting for nearly 50% of the global plasma proteins market (cf. 10–15% each for plasma‐derived Factor VIII and albumin), but their recovery (by multiple precipitation steps followed by ion exchange chromatography) incurs large losses during precipitation.^3^, ^10^, ^11^ In recognition of this, and in response to increasing market demands for polyclonal immunoglobulins as biotherapeutics, new approaches (largely chromatographic), able to simultaneously improve product recovery, purity and productivity, are gradually replacing the three‐step ethanol fractionation process.^3^, ^10^, ^11^

Ion exchange is the preferred chromatographic technique for commercial recovery and purification of polyclonal antibody therapeutics from human plasma.^10^ Protein A affinity chromatography, the lynchpin purification technique in industrial manufacture and production of clinical grade monoclonal antibodies,^12^ is not employed and neither is Protein G affinity chromatography.^3^, ^10^ Protein A fails to bind all human IgG subclasses, particularly IgG_3_, which contributes to the humoral viral defence. Protein G on the other hand binds all subclasses of IgG, but the strongly acidic elution conditions required are too harsh for a subpopulation of polyclonal human IgG.^10^ Additional negating factors for chromatography on Protein A/G columns are potential ligand leaching^10^ concerns, and unacceptably high media costs^10^, ^13^ for purifying huge quantities of polyclonal antibodies (global production of intravenous immunoglobulins reached 140 tonnes in 2014^13^).

Though well adapted to the fractionation and polishing of pre‐purified plasma fractions and processing of monoclonal antibody culture supernatants, fixed bed chromatography is less well suited to product capture from much more complex plasma and serum feeds.^9^, ^14^ The presence of troublesome fouling components within these feeds, especially suspended lipoproteinaceous materials, can severely compromise chromatographic operations; the extent to which this occurs is largely governed by the nature of the immobilised ligand and that of base matrix to which it is attached.^5^, ^6^, ^15^

The requirement for extensive clarification before loading biological feedstocks on fixed bed chromatography columns is essential, as suspended solids within them become trapped in the interstitial space between media particles, rapidly cutting off liquid flow through the bed and posing unacceptable demands on CIP/SIP. In stark contrast, modern direct capture techniques, such as expanded bed adsorption and high‐gradient magnetic fishing (HGMF), are suited to fast recovery of target species from large volumes of unclarified biological liquors,^15^, ^16^, ^17^, ^18^ afford potential improvements in product yield, productivity and process efficiency cf. conventional clarification followed by fixed bed chromatography, and importantly, both methods have been successfully applied for product recovery from plasma/serum feeds.^9^, ^14^ HGMF, a scaleable technique combining adsorption of a product of interest onto low‐cost functionalised magnetic particles, with subsequent retrieval and processing of the product laden magnetic support by means of high‐gradient magnetic separation (HGMS) technology,^18^, ^19^, ^20^, ^21^ affords a fast and efficient means for isolating protein products from crude feedstocks.^9^, ^15^, ^18^, ^21^, ^22^, ^23^, ^24^, ^25^, ^26^, ^27^

Here we describe systematic development of an HGMF process for the capture and purification of polyclonal antibodies from an unclarified rabbit antiserum feedstock, employing non‐porous superparamagnetic adsorbents functionalised with the hydrophobic‐charge induction ligand, 4‐mercaptoethylpyridine (4‐MEP). The choice of 4‐MEP as ligand was informed by a previous study^6^ that compared the suitability of eight commercial chromatography media intended for antibody purification, for capture and purification of polyclonal immunoglobulins from complex clarified rabbit antiserum feeds. Of the five low molecular class synthetic ligand based media tested, only the hydrophobic charge induction matrix MEP HyperCel compared favourably with the much more expensive rProtein A based media.

Four different activation routes were employed for anchoring 4‐MEP to a favoured magnetic support particle. The resulting adsorbents were subsequently screened in small‐scale magnetic rack tests for their ability to purify immunoglobulins from a clarified antiserum feedstock, and the best of these was selected for all further work. Subsequently conditions for optimal use of the chosen MEP‐linked magnetic adsorbent for immunoglobulin capture and purification were systematically established, and the adsorbent holding capacity of a small high‐gradient magnetic filter unit positioned in the bore of a mini‐pilot scale ‘ON–OFF’ permanent HGMS unit was determined. Efficient use of MEP‐linked magnetic adsorbents was then demonstrated at sevenfold increased scale in an HGMF process to recover immunoglobulin in substantially pure form and high yield from a ‘dirty’ i.e. unclarified, antiserum feed. The study concludes with a discussion of some of the measures required to maximise HGMF's untapped potential as a direct capture technology for fast recovery of target antibodies and other proteins expressed at high titre in complex unclarified bioprocess liquors.

## EXPERIMENTAL

### Materials

The 430 stainless steel wire matrix (KnitMesh type 9029) and the rabbit anti‐human transferrin antiserum employed in this work were received as gifts from KnitMesh Ltd (South Croydon, Surrey, UK) and Dako Agilent Pathology Solutions (Glostrup, Denmark), respectively. Iron (II) chloride hexahydrate, dimethyl sulphoxide (99.5%) and Silica gel 60 F_254_ plates for thin‐layer chromatography (TLC) were supplied by Merck (Darmstadt, Germany), while iron (III) tetrahydrate was purchased from Mallinckrodt Baker B.V. (Deventer, the Netherlands). The following materials were purchased from the Sigma‐Aldrich (St. Louis, MI, USA): thiolacetic acid; 4‐vinylpyridine; diethyl ether; sodium bicarbonate; sodium chloride; anhydrous magnesium sulphate; hydrochloric acid; isopropanol; deuterium oxide; methanol; glacial acetic acid; 3‐aminopropyltriethoxysilane; glycerol; glutaraldehyde (50%, photographic grade); sodium borohydride; sodium carbonate; epichlorohydrin (ECH); allyl bromide (AB); allyl glycidyl ether (AGE); divinyl sulphone (DVS); N‐bromosuccinimide (NBS); Trizma® base (≥99%); ammonium sulphate; citric acid monohydrate; sodium citrate dihydrate; anhydrous sodium acetate; and, protein standard (bovine serum albumin). All the reagents for the rabbit immunoglobulin immunoturbidimetric assay (i.e. dilution buffer – S2005; reaction buffer – S2008; goat anti‐rabbit immunoglobulins ‘GoaRbIg’ – Z0421; dilution buffer for GoaRbIg – TO 0463; and Ig standard – X0903 concentrate), were obtained from Dako Agilent Pathology Solutions (Glostrup, Denmark). Bicinchoninic acid (BCA) protein assay kits were supplied by Pierce (Rockford, IL, USA), while pre‐cast Invitrogen branded SDS‐PAGE gels, Novex Colloidal Blue protein staining kit, molecular weight markers (myosin – 200 kDa, β–galactosidase – 116.3 kDa, phosphorylase b – 97.5 kDa, bovine serum albumin – 66.2 kDa; glutamate dehydrogenase – 55.4 kDa, ovalbumin – 45 kDa, carbonic anhydrase – 31 kDa, trypsin inhibitor – 21.5 kDa, lysozyme – 14.4 kDa; aprotinin – 6.5 kDa), sample and running buffers were acquired from Thermofisher (Waltham, MA, USA). All other materials not identified above were acquired from Sigma‐Aldrich and Merck.

### Synthesis of 4‐mercaptoethylpyridine hydrochloride

The hydrophobic‐charge induction ligand used in this work, 4‐mercaptoethylpyridine HCl, was prepared using a modified version of the method described by Burton.^28^ Under stirring 125 mL of 4‐vinylpyridine (95%) was pre‐chilled to –30°C in a methanol/dry ice bath. Thiolacetic acid (85 mL) was then added at a rate of 0.5 mL per min and the temperature was maintained at –23°C by immersing the reaction vessel in a water bath held at 20 ± 2°C. After 15 h stirring at room temperature, the product was mixed with 200 mL of diethyl ether and extracted four times with 160 mL portions of saturated sodium bicarbonate solution. The separated ether layer was subsequently washed twice with 150 mL portions of saturated sodium chloride, treated with activated charcoal to reduce colour, dried over anhydrous magnesium sulphate, then filtered and evaporated under vacuum (bath temperature ∼30°C). The resulting oil was stirred with 400 mL of 6 mol L^‐1^ HCl. After 4 h the acid layer was reduced under vacuum, the dried residue was re‐slurried with 150 mL of isopropanol and recrystallised overnight at –18°C, before filtering and finally drying under vacuum to yield a dark creamy solid. This was then re‐dissolved in another 400 mL portion of 6 mol L^‐1^ HCl and stirred at room temperature. The reaction was monitored by TLC and stopped after 40 h. Subsequent concentration, re‐crystallisation from 150 mL of isopropanol, filtration and drying gave a creamy white solid (81 g, 40% yield) identified as the desired compound: ^1^H NMR (D_2_O, 300 MHz) δ 2.89 (t, 2 H, C*H*_*2*_‐SH, *J* 7.1 Hz), 3.18 (t, 2 H, Pyr‐C*H*_*2*_, *J* 7.1 Hz), 7.89 (d, 2 H, H‐2, H‐2′, Pyr, *J* 6.6 Hz), 8.59 (d, 2 H, H‐3, H‐3′, Pyr, *J* 6.8 Hz); ^13^C NMR (D_2_O, 300 MHz) δ 23.27 (*C*H_2_‐SH), 39.22 (Pyr‐*C*H_2_), 127.85 (Pyr, C‐3, C‐3′), 140.64 (Pyr, C‐2, C‐2′), 162.44 (Pyr, C‐4).

### Support handling

A strong (>0.5 T) NdFeB permanent magnet block (Danfysik A/S, Jyllinge, Denmark) was used to separate magnetic particles from liquid phases during preparation of the magnetic adsorbents (10 g scale). In small‐scale functionalisation steps and in batch binding studies, magnetic adsorbents were recovered from suspension with the aid of ∼0.15 T side‐pull NdFeB racks (chemagic Stands 50 k Type A and 2 × 12, PerkinElmer chemagen Technolgie GmbH, Baesweiler, Germany).

### Manufacture of 4‐mercaptoethylpyridine‐linked superparamagnetic adsorbents

**Figure 1:**
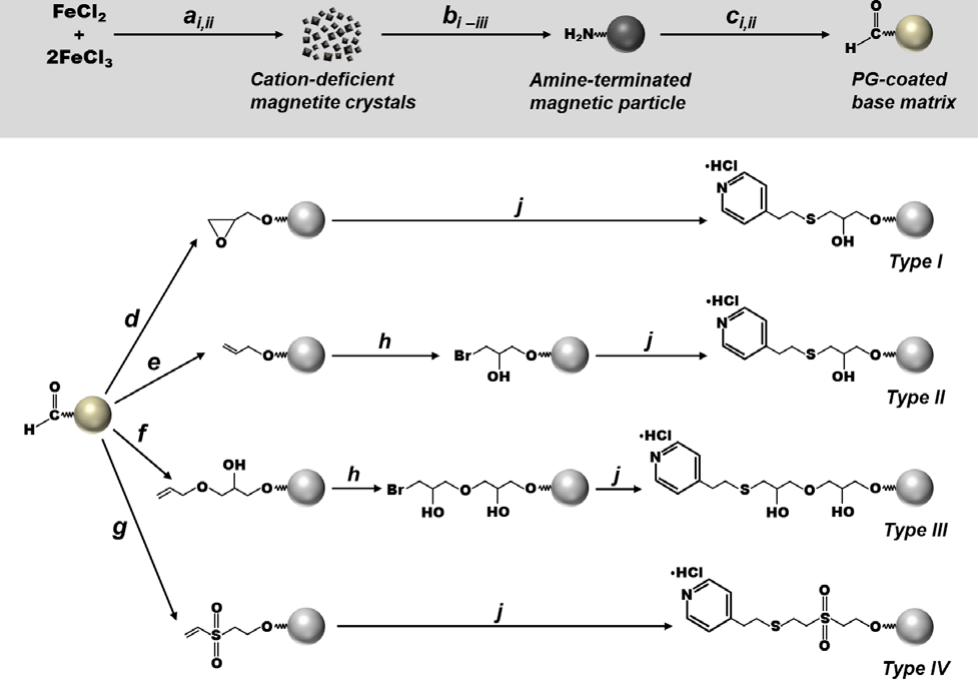
Scheme for the manufacture of the 4‐MEP linked magnetic supports used in this study. Key: **a**_i_ NaOH, rt; **a**_ii_ washing (water → NaCl → methanol), rt; **b**_i_ 3‐aminopropyltriethoxysilane, glacial acetic acid, methanol, 600 s @ 13 000 rpm → 2 h @ 6000 rpm, rt; **b**_ii_ glycerol, N2, 11 h @ 110 °C, 0.5 h @ 160 °C; **b**_iii_ washing (water → NaCl → water), rt; **c**_i_ glutaraldehyde, pH 11, 1 h, rt; **c**_ii_ washing (water → NaCl → water); **d** epichlorohydrin, NaOH, NaBH_4_, 6 h, rt; **e** allyl bromide, NaOH, NaBH_4_, DMSO, 18–20 h, rt; **f** allyl glycidyl ether, NaOH, NaBH_4_, DMSO, 18–20 h, rt; **g** divinylsulphone, Na_2_CO_3_, 1 h, rt; **h** N‐bromosuccinimide, 1 h, rt; **j** 4‐mercaptoethyl pyridine hydrochloride, Na_2_CO_3_, NaBH_4_, 48 h, rt.

The construction of MEP‐linked magnetic adsorbents employed in this study is shown schematically in Fig. 1 and described below.

#### Base materials

Detailed steps for the preparation of polyglutaraldehyde (PG) coated magnetic starting materials have been presented in full elsewhere^20^, ^29^, ^30^ and are only briefly summarised here. 10 g of cation deficient superparamagnetic iron oxide crystals were prepared by chemical precipitation involving aqueous mixed iron chloride salts and a strong base. These crystals were subsequently formed into submicron‐sized particles by performing silanisation with 3‐aminopropyltriethoxysilane (3‐APTES) in a high‐shear environment, and then stabilising the aminosilane coat by curing the magnetic particles in glycerol at high temperature under nitrogen.^20^ The resulting amine‐terminated superparamagnetic particles were then coated with a layer of polyglutaraldehyde by stirring with 2% (v/v) glutaraldehyde at pH 11 in a pH stat vessel^29^ to yield irregular particles (D_v50_ = 0.80 µm, D_v20–80_ of 0.65–1.12 µm) with high saturation magnetisation (M_S_ = 53.3 ± 2.1 Am^2^ kg^‐1^) and low remanence (M_R_ = 0.28 ± 0.09 Am^2^ kg^‐**1**^).

#### Activation

Next, PG‐coated supports were variously activated (as described previously by Heebøll‐Nielsen *et al*.^23^, ^30^) before coupling with 4‐MEP to produce the four different MEP‐linked supports designated Types I–IV (Fig. 1). The manufacture of type I supports involved prior activation of PG‐coated particles (25 g L^‐1^) for 6 h at 21°C with 5% (v/v) epichlorohydrin (ECH) in 0.5 mol L^‐1^ NaOH and 19 mmol L^‐1^ NaBH_4_. The preparation of type II and III MEP‐linked supports involved activation with allyl bromide (AB) and allyl glycidyl ether (AGE), respectively, using a procedure adapted from Burton and Harding.^31^ PG‐coated supports (33 g L^‐1^) suspended in 0.15 mol L^‐1^ NaOH and 36 mmol L^‐1^ NaBH_4_ in 50% (v/v) DMSO were mixed with AB (type II) or AGE (type III) to a final concentration of 50% (v/v), incubated for 48 h at room temperature and then washed extensively with water. The resulting allylated particles (20 g L^‐1^) were reacted (1 h, 21°C) with 0.14 mol L^‐1^ *N*‐bromosuccinimide (NBS) introducing reactive bromohydrin moieties. Finally, the creation of type IV magnetic adsorbents involved DVS‐activation. In this procedure, DVS was added at regular intervals over 600 s to PG‐coated support particles (25 g L^‐1^) in 0.5 mol L^‐1^ Na_2_CO_3_ containing 18 mmol L^‐1^ NaBH_4_ to a final amount of 8 mL g^‐1^ particles. The reaction was subsequently allowed to proceed for 1 h at room temperature.

#### Coupling

Before coupling, all activated supports were washed extensively with water by repeated cycles of resuspension, mixing and magnetic separation. The pH of a 0.7 mol L^‐1^ solution of 4‐mercaptoethylpyridine HCl in water was adjusted to 11.5 with saturated NaOH and subsequently diluted to 55 mmol L^‐1^ in 0.5 mol L^‐1^ Na_2_CO_3_ containing 28 mmol L^‐1^ NaBH_4_. Portions of this solution were then mixed with ∼1 g quantities of activated supports (final support concentration of 6 g L^‐1^) in sealed glass Duran® bottles for 48 h on a vibrating shaker at 21°C. The finished supports were magnetically retrieved from suspension and washed copiously with 0.5 mol L^‐1^ NaCl and then water before finally storing at 4°C in 20 mmol L^‐1^ sodium phosphate, 1 mol L^‐1^ NaCl, pH 6.8 until required. The presence of 4‐MEP in the final adsorbent preparations was assessed by FTIR.

### Feedstock

The rabbit antiserum used in this work is a highly complex feedstock containing unusually high levels of immunoglobulin (Ig).^5.6^ For all small‐scale binding studies and determination of adsorbent loading capacity to use in HGMF, crude rabbit antiserum pools from ‘Danish Whites’ were first filtered free of particulate matter by passage through a Nalgene disposable dead end membrane to yield clarified undiluted sera hereafter designated ‘100% serum strength (the mean Ig and total protein concentrations in serum were determined as 25 ± 1.3 g L^‐1^ and 93 ± 2 g L^‐1^, respectively, and the electrical conductivity at 20°C was 10.8 mS cm^‐1^). This feedstock was used in variously diluted forms yielding serum strengths ranging from 0.04 to 22.5% (v/v). For the recovery of Ig by HGMF, the crude (unclarified) antiserum was simply diluted tenfold.

### Batch binding and elution studies

Small‐scale batch binding and elution tests were conducted in 2 mL screw‐capped vials (Sarstedt, Nümbrecht, Germany) at room temperature. Supports (1.8–45 mg) were magnetically recovered from storage buffer, resuspended and equilibrated in a defined volume of appropriate binding buffer by two cycles of resuspension and magnetic separation, before portioning into vials. Some samples were mixed with 1.5 mL aliquots of diluted antiserum for various times at 21°C on an IKA VXR‐S17 vibrating shaker platform (IKA Labortechnik, Staufen, Germany), whereas others were sacrificed for dry weight measurements in order to determine the exact amounts of support used in each test. After binding, supports were retrieved on a magnetic rack, washed once briefly (30 s) with binding buffer, before adding elution buffer (0.1–1 mol L^‐1^ sodium acetate) and incubating for 600 s on a vibrating shaker. In most cases two sequential elution cycles were performed.

The initial selection of buffers for equilibration/washing and elution was informed by previous work on the chromatography of human^32^ and especially rabbit^5^, ^6^ polyclonal antibodies on MEP HyperCel.

In preliminary screening of the various MEP‐linked support types (I–IV), supports (9–12 mg) were equilibrated with 50 mmol L^‐1^ Tris–HCl pH 8 and then incubated with 1.5 mL of 20% (v/v) antiserum for 0.5 h. After magnetic separation the supports were washed with binding buffer, before finally incubating protein laden adsorbents for 0.5 h with 1.5 mL of elution buffer (50 mmol L^‐1^ sodium acetate, pH 4).

All subsequent characterisation of binding, washing and elution operations were conducted with type III supports. Binding optimisation was performed at 1.5 mL scale (using 10% v/v antiserum feedstocks and 8.5 mg mL^‐1^ of supports) by systematically varying the contact time (20–900 s; 50 mmol L^‐1^ Tris–HCl pH 8), pH (employing 50 mmol L^‐1^ sodium citrate pH 6 and 50 Tris–HCl pH 7–9 buffer) and doping of binding buffer (50 mmol L^‐1^ Tris–HCl pH 8) and diluted antiserum with various concentrations (0–150 mmol L^‐1^) of ammonium sulphate or sodium chloride. After identifying the ‘best’ conditions for Ig binding, the adsorption performance of the type III MEP‐linked support was characterised further in two sets of experiments in which various dilutions (4.5‐ to 2500‐fold) of antiserum were contacted with a fixed concentration of supports (4.6 mg mL^‐1^), and 10.2% (v/v) antiserum was mixed with support at final concentrations of 1.2–30 mg mL^‐1^. The key variable examined in optimisation of washing immediately post‐binding and prior to elution was the support concentration, whereas for elution, in addition to varying support concentration, different strengths (0.05–1 mol L^‐1^) of sodium acetate and citrate buffers and fine tuning of elution pH (between 3.5 and 4.5) were explored.

Liquid‐phase samples from all of the above tests (antiserum feedstock, unbound, wash and elution fractions) were retained for determination of residual Ig and total protein contents, and protein composition. The amounts of bound Ig and total protein were computed from the difference in liquid phase concentration before and after binding, and in some cases adsorption data were fitted to the Langmuir model^33^ (Equation (1)):

(1) $$Q^{*}=Q_{\max}\frac{C^{*}}{K_{d}+C^{*}}$$

where Q* and C*, respectively, represent the equilibrium concentrations of adsorbed and liquid‐phases binding species, *Q*_*max*_ is the maximum protein binding capacity of the support, and *K*_*d*_ is the dissociation constant. Data was fitted to the model using the *χ* ^2^ minimisation procedure of OriginPro 2017 software (OriginLab Corporation, Northampton, MA, USA).

### High‐gradient magnetic fishing (HGMF)

#### Equipment set‐up and operation

**Figure 2:**
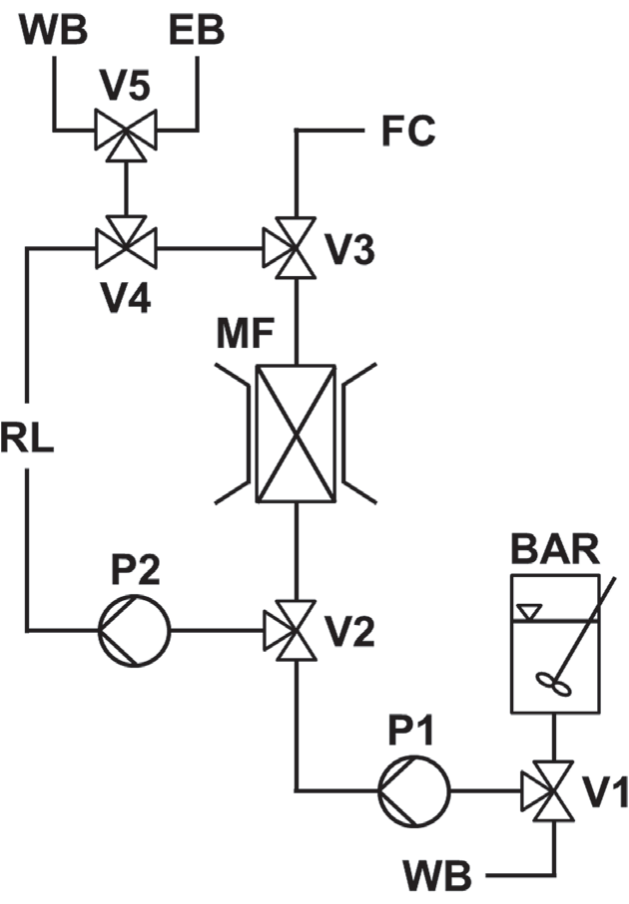
Schematic representation of the HGMF system employed. Key: batch adsorption reactor (BAR); magnetic filter (MF); fraction collector (FC); valves (V1–V5); pumps (P1 & P2); wash buffer (WB); elution buffer (EB).

A schematic illustration of the laboratory scale HGMF rig employed in this work is shown in Fig. 2; the operation of which has been described in detail by Meyer *et al*.^25^ At its core is a 70 kg mini‐pilot scale cyclically operated ‘ON–OFF’ permanent magnet based high‐gradient magnetic separator (HGF‐10, Steinert Elektromagnetbau GmbH, Köln, Germany) with an adjustable air‐gap between the poles of 1.5–2.5 cm. In this study, the gap was set to 1.5 cm and the measured magnetic flux densities in the ‘ON’ and ‘OFF’ positions were, respectively, 0.56 and 0.03 T. A small magnetic filter was constructed by inserting a tightly‐rolled mat of woven 430 stainless steel mesh (fibre thickness ∼110 µm) into a 4.4 mL plastic canister (56 mm long × 10 mm i.d) so that it occupied 11% of the working volume (i.e. voidage = 0.89, void volume = 3.9 mL). The resulting magnetic filter was then positioned vertically between the pole shoes (area = 100 mm × 80 mm). The HGMF set up comprised: (i) a stirred batch adsorption reactor; (ii) the aforementioned magnet and magnetic filter canister; (iii) two peristaltic pumps (Masterflex L/S Easy‐Load model 7518‐00, Cole Parmer Instruments Co., Vernon Hills, IL, USA); and (iv) SuperFrac fraction collector fitted with high flow adaptors (GE Healthcare, Uppsala, Sweden). The flow paths for particle loading, washing, protein elution, and particle recovery were controlled with a set of three‐way solenoid switching valves (Bürkert Werke GmbH Fluid Control Systems, Ingelfingen, Germany). With the field switched ‘ON’, the adsorbent particle/feedstock suspension was pumped to the magnetic filter through values 1–3 via pump 1. Washing and elution operations were conducted with the aid of a recycle loop (11.6 mL). After filling the loop via valves 4 and 5, the canister–loop circuit (available volume = 15.5 mL) was closed by switching valves 2 and 3. The field was switched ‘OFF’ and the liquid contained within the closed loop was driven at high speed via pump 2 to release particles from the magnetic filter wires. Subsequently the field was switched back ‘ON’ to recapture the magnetic adsorbent particles, and after turning valve 3, washed off or eluted materials were sent to the fraction collector. Finally, support particles were recovered by switching the field ‘OFF’ and pumping out of the system via pump 1. The HGF‐10 magnet, pumps and valves were all controlled by National Instruments™ LabVIEW software (Austin, TX, USA).

#### Determination of filter capacity

Prior to carrying out HGMF recovery of Ig from unclarified rabbit antiserum, the loading capacity of the HGMF filter for type III MEP‐linked magnetic adsorbents was examined in a breakthrough study employing clarified 10% (v/v) antiserum containing support particles at a concentration of 30 mg mL^‐1^. With the field switched ‘ON’ the suspension was loaded into the magnetised filter at a linear flow rate of 24 m h^‐1^. Particle breakthrough in the filter effluent was monitored by gravimetric measurement of the particle mass in collected samples.

#### Recovery of immunoglobulins from unclarified rabbit antiserum by HGMF

Type III magnetic MEP‐linked adsorbents (previously equilibrated in 50 mmol L^‐1^ Tris–HCl, pH 8) were resuspended in crude unclarified rabbit antiserum (diluted in the same buffer) to give final particle and antiserum concentrations of 31.7 mg mL^‐1^ and 10% (v/v), respectively, and thereafter mixed at room temperature with an overhead stirrer for 600 s. Subsequently, with the magnet switched ‘ON’, the particle/antiserum suspension was pumped upward through the magnetic filter at a linear flow rate of 24 m h^‐1^. Pumping was stopped before breakthrough was expected, i.e. after 11.5 mL of suspension containing 365 mg particles had been loaded into the magnetised filter. The recycle loop (11.6 mL) was then filled with washing buffer (50 mmol L^‐1^ Tris–HCl, pH 8) and after turning the field ‘OFF’, the suspension was pumped around the recycle loop upwards with respect to the magnetic filter at a velocity of 92 m h^‐1^ for 60 s, to wash out entrained and/or loosely adsorbed materials. The particles were subsequently recaptured by switching the field back ‘ON’, the flow rate was lowered to 24 m h^‐1^ and the washings were pumped out of the rig to the fraction collector. Bound Ig and protein was desorbed from the retained MEP‐linked adsorbent particles in 600 s elution cycles in exactly the manner just described for washing, i.e. by filling the recycle loop with elution buffer (0.5 mol L^‐1^ sodium acetate, pH 4), and rapidly circulating the particles around the closed system loop. The volumes of all collected fractions were accurately measured and all fractions were analysed for immunoglobulin and total protein contents, and composition by SDS‐PAGE.

### Analytical techniques

Thin‐layer chromatography was performed on Merck Silica gel 60 F_254_ plates and spots were visualised under ultra‐violet light. ^1^H and ^13^C NMR spectra were recorded at 500 and 125.7 MHz, respectively, on a Varian Inova 500 spectrometer.

For qualitative FT‐IR analysis of solid supports, 2 mg samples previously dried in a desiccator were mixed with 298 mg potassium bromide, ground down to a fine powder and hydraulically pressed (15 tonnes) into tablet form. Each tablet was subjected to 64 scans (averaged at a resolution of 2 cm^−1^) in a Nicolet 380 FT‐IR (Thermo FisherScientific, Waltham, MA, USA) in direct beam mode.

Magnetic particle content was determined using a dry weight method based on that described by Hubbuch and Thomas.^20^ Bulk magnetic properties were investigated at ambient temperature in a MicroMag™ 2900 Alternating Gradient Magnetometer (PMC, Princeton, NJ, USA) and particle size analysis was performed with a Mastersizer2000 particle size analyser (Malvern Instruments Ltd, Malvern UK).

The concentration of antibodies in bulk phase samples was determined using a robust high throughput immunoturbidimetric assay advanced by Bak and co‐workers.^34^ The method, based on the scattering of light caused by the formation of different sizes of immune complexes by different ratios of antibody to antigen, was specifically developed for in‐process determination of polyclonal antibody concentration in crude samples, and has been shown to be insensitive to all of the equilibration/binding/wash and elution buffer combinations employed in this work. The original 96‐well plate procedure was adapted so that it could be performed automatically in a spectrometric robot system (Cobas Mira Plus Random Access Analyser, Roche Diagnostic Systems, Rotkreutz, Switzerland) as follows: Samples (35 µL) were mixed with 126 µL of reaction buffer and incubated at 37 °C. After 300 s, the absorbance was recorded at 340 nm and 84 µL of twofold diluted GoaRbIg was added. After 300 s incubation at 35 °C, the absorbance was again recorded at 340 nm. Standards of purified immunoglobulins from non‐immunised rabbits, prepared in dilution buffer to a final concentration ranging from 6.6 to 500 µg mL^‐1^, were treated in exactly the same way as the samples.

The total protein contents of liquid phase samples were determined by the BCA protein assay (Pierce Rockford, IL, USA) adapted for use in the Cobas Mira Analyser. All results are expressed in mg bovine serum albumin (BSA) equivalents. Corrections for variation in Ig content in samples were not applied. In the assay the $A_{562\mathrm{nm}}^{0.1\%}$ rabbit IgG: $A_{562\mathrm{nm}}^{0.1\%}$ BSA ratio = 1.12; thus calculated figures for Ig purity, purification and yield factors, in this work, are underestimated.

Protein composition was analysed by reducing SDS‐PAGE^35^ in NuPAGE® Novex Bis‐Tris (4–12%) gels. Images of gels were captured using a GelDoc2000 system (Bio‐Rad Laboratories, Hercules, CA, USA) and the relative densities of stained bands in destained gels were analysed using ImageJ software, downloaded from http://rsb.infonih.gov.ij/.

The RSA contents in eluates are expressed as percentages of the Ig signal by dividing the RSA band intensity in each lane by the combined Ig heavy and light chain band intensities, and then multiplying by 100.

## RESULTS AND DISCUSSION

### Magnetic support design

Gu and coworkers^36^ recently demonstrated effective use of magnetic agarose based adsorbents derivatised with the hydrophobic‐charge induction ligand, 5‐aminobenzimidazole, for antibody capture from a dilute mimetic serum (IgG + BSA), mimetic serum ‘spiked’ with yeast cells, and CHO culture supernatant. The much more complex, concentrated and fouling antiserum feeds used in this work dictated choice of a less ‘challenged’ adsorbent design. Much previous work confirms that sub‐micron sized non‐porous magnetic adsorbents fashioned from the PG‐coated superparamagnetic base particle described by Hubbuch and Thomas^20^ are well suited for operation in unclarified and heavily fouling bioprocess liquors.^18^, ^22^, ^23^, ^24^, ^25^, ^30^ It is rarely appreciated that non‐porous supports are less prone to fouling and easier to clean once fouled, than their porous counterparts,^37^, ^38^ and are therefore inherently more useful for product capture and purification from fouling liquors.^15^ For porous supports, intra‐particle pore fouling is an especially serious issue. Foulants ingressing into and trapped within pores are more difficult to dislodge that those adhering to the external surface; this is largely because internal pores are effectively isolated from the effects of external fluid shear cf. the external surface.^37^, ^38^

### Screening of MEP‐linked adsorbents prepared via different activation routes

**Figure 3:**
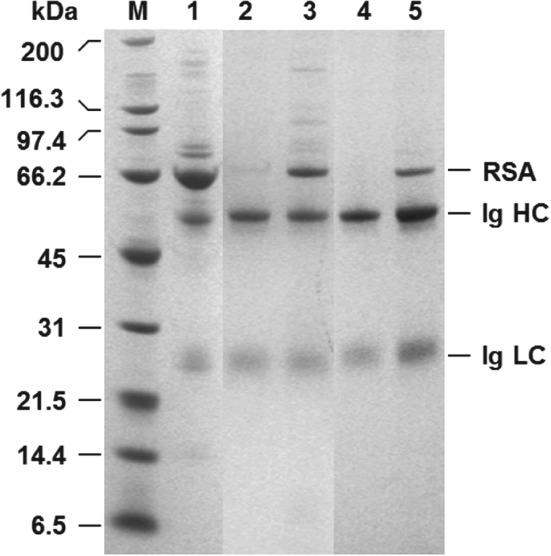
Reducing SDS‐PAGE analysis of eluates from MEP‐linked supports obtained during Ig recovery from clarified rabbit antiserum. Key: molecular weight markers (M); rabbit antiserum (1); eluates from MEP support types I (2), II (3), III (4) and IV (5); RSA = rabbit serum albumin; Ig = immunoglobulin; HC = heavy chain; LC = light chain.

Four different activation chemistries were employed on PG‐coated magnetic particles prior to coupling MEP (Fig. 1). FT‐IR analysis confirmed successful installation of 4‐MEP in all cases. The spectra of finished adsorbents contained absorbance peaks at ∼804 cm^‐1^ and ∼1599 cm^‐1^ corresponding to the thioether bond (‐C‐S‐C‐) and imine, respectively, whereas those of controls (i.e. activated supports subjected in parallel to the same coupling conditions, but without 4‐mercaptoethylpyridine) showed no such peaks. Table 1 shows the results of initial matrix scouting performed with the various MEP‐linked supports (I–IV) at a concentration of 7 mg mL^‐1^ in 20% (v/v) clarified rabbit antiserum representing an effective challenge of 28.6 mL of undiluted ‘100%’ serum per g support. Under these conditions, between 5.6 and 25.4% of the available Ig and 9.1 to 19.8% of the total soluble protein was removed from antiserum. Only two of the four supports bound Ig selectively over total protein, i.e. types I and III. Though the highest Ig binding (181 mg g^‐1^) was demonstrated by the type I support prepared by ECH activation route with purification, the type III support (Ig binding capacity = 170 mg g^‐1^), produced via two‐step AGE activation and bromination procedure, demonstrated substantially greater Ig binding selectivity. The purity on adsorption was >56% (cf. 34.4% for type I) reflecting a purification adsorption (PF_ads_) of 2.1 (cf. <1.3 for type I). Surprisingly, type II and IV supports displayed greater preference for the binding of non‐Ig proteins reflected by lower purity of Ig in the adsorbed state, i.e. 11.5% and 18.5% for types II and IV respectively cf. that of the initial antiserum (26.9%). In all cases, the wash step, conducted immediately post‐binding, desorbed large amounts of entrained and weakly adsorbed protein, such that significant enhancements in adsorbed Ig purity were achieved prior to elution. For example, in the best case (type III supports) 46% of the adsorbed total protein was desorbed at the expense of a 14% loss in bound Ig such that the purity rose to 89%. In the subsequent pH elution step, Ig was preferentially eluted over other adsorbed proteins from all supports (Table 1). A palpable trend, supported by electrophoretic analysis (Fig. 3), is that the greater the selectivity displayed by the support (type III > I > IV > II) during the binding step, the higher the Ig purity of the final eluate (95% for type III, 92% for type I, 83% for type IV and 64% for type II). All eluates contained rabbit serum albumin (RSA), the main impurity in serum, but the levels of RSA contamination varied markedly. Whereas strong RSA bands are evident in lanes corresponding to eluates from type IV and II supports, they are barely discernible in the eluates from the type I and III adsorbents. With the aid of Image J analysis, the signal intensities for RSA expressed as percentages of the combined Ig band intensities in each lane were determined as 2, 4, 13 and 41% for the eluates from support types III, I, IV and II, respectively. The type III adsorbent prepared by the AGE activation route was selected for use in all further studies in view of its superior overall performance. Nearly 14% of the Ig present in the antiserum was recovered with a purity of 95% representing a purification of >3.5‐fold.

**Table 1:** Comparison of MEP linked support types I – IV (see Fig. 1) for the recovery of immunoglobulins from 20% (v/v) clarified rabbit antiserum (see text for details)

Support ID	Activation method	Binding						Elution		
		Ig bound (%)	Total protein bound (%)	Q*_Ig_ (mg g^‐1^)	Q*_Total protein_ (mg g^‐1^)	Adsorbed Ig purity (%)	PF_ads_ *	Ig yield (%)	Ig purity (%)	PF**
**Type I**	ECH	25.4	19.8	181	527	34.35	1.28	12.55	91.7	3.41
**Type II**	AB	5.6	13.1	40	347	11.53	0.43	2.93	63.9	2.38
**Type III**	AGE	23.8	11.4	170	303	56.11	2.09	13.7	95.0	3.53
**Type IV**	DVS	12.5	18.1	89	480	18.54	0.69	10.82	83.3	3.10

* PF_ads_ = purification factor for the adsorption step and

** PF = overall purification factor.

Though neither the extent of activation nor MEP ligand density were measured in this work, the differences in Ig binding and purification performance noted here (Table 1 and Fig. 3) for the various MEP‐linked magnetic adsorbents likely stem from a complex interplay of spacer chemistry (Fig. 1) and immobilised ligand density. Boschetti^39^ stressed: (i) the potential benefits of including a sulphur atom in the spacer (applicable in the case of the type IV support prepared via DVS activation, see Fig. 1); (ii) the importance of employing sufficiently hydrophobic spacer arms; and crucially (iii) that IgG adsorption is strongly dependent on the density of hydrophobic MEP ligands anchored to the support's surface. Adsorption can only occur when a certain critical hydrophobicity^40^ is reached (in the case of MEP HyperCel Boschetti^39^ states this is >40 mmol mL^‐1^); beyond this point binding capacity increases until saturation. The superior performance of the type III adsorbent over other types in this work likely reflects that it represents the best ‘ligand density/spacer hydrophobicity’ combination.

### Optimisation of binding conditions

**Figure 4:**
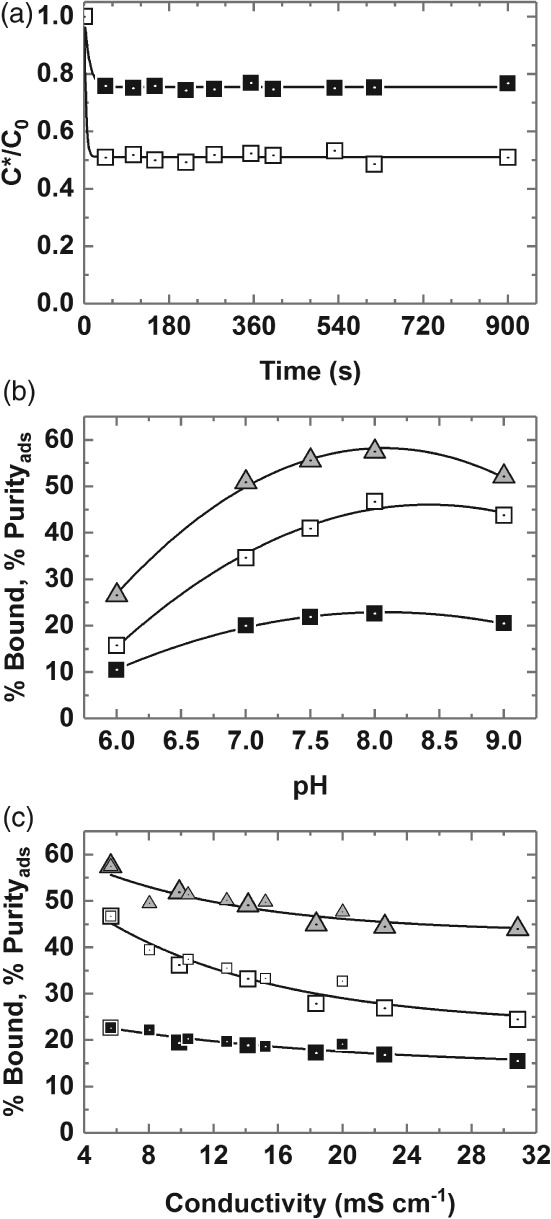
Optimisation of conditions (a – time, b – pH, c – conductivity) for the binding of rabbit Ig from clarified 10% (v/v) antiserum on type III MEP‐linked magnetic adsorbent particles (fixed support concentration = 8.5 mg mL^‐1^). Key: percentage bound Ig (white squares); percentage bound protein (black squares); percentage Purity_ads_ (grey up‐triangles). The large and small symbols used in plot c are for data obtained with ‘added NH_4_(SO_4_)_2_’ and ‘added NaCl’ respectively.

Figure 4 shows the results of systematic experiments aimed at identifying effective binding conditions for selective recovery of immunoglobulins from clarified 10% (w/w) rabbit antiserum using type III non‐porous magnetic MEP‐linked adsorbents at support concentration of 8.5 mg mL^‐1^ (challenge = 11.8 mL equivalents of 100% serum per g support).

#### Kinetics

The kinetics of Ig and protein binding are presented in Fig. 4(a). As has been noted before with other adsorbents fabricated from the same submicron sized and essentially non‐porous magnetic base matrix, binding equilibrium is attained very rapidly;^22^, ^25^, ^26^, ^30^, ^41^, ^42^ in the present case by the first time point, i.e. 45 s, which represents the minimum time required for handling samples on the magnetic rack. No difference in binding kinetics for Ig and total protein was observed in the experiment, thus the adsorbed Ig purity remained constant averaging 55.34 ± 2.38% over 10 time points between 45 and 900 s. For purely practical reasons, a binding time of 600 s was adopted in all subsequent work.

#### pH

Varying the pH of the binding buffer used for support equilibration and dilution of the antiserum exerted a strong impact on both the amount and selectivity of Ig binding by the adsorbent (Fig. 4(b)). Maximum Ig binding (46.7% of that presented) and selectivity of adsorption (PF_ads_ = 2.07–2.14 corresponding to a purity on adsorption of 57.5%) were obtained at a pH of 8, and substantial retention of binding selectivity was maintained between pH 7 and 9. However, shifting to lower pH values seriously compromised both the level and selectivity of Ig binding. For example, at pH 6 < 16% of the available Ig was adsorbed and all binding selectivity was lost (calculated Ig purity of 26.6% is less than that of the initial antiserum). The pH dependence of rabbit Ig adsorption on type III MEP‐ magnetic adsorbents observed here accords with earlier studies conducted with human polyclonal IgG and MEP HyperCel, and is consistent with the 4‐MEP ligand's pKa of 4.8 and the pH‐dependent adsorption–desorption mechanism described by Burton and Harding^31^ and corroborated by Boschetti and colleagues,^32^, ^39^ i.e. hydrophobic interaction under near physiological conditions in the absence of a lyotropic salt, and desorption via pH induced electrostatic charge repulsion.

#### Influence of salt concentration

Guerrier *et al*.^32^ previously reported that polyclonal human IgG binding to MEP HyperCel is salt‐independent up to an electrical conductivity of 100 mS cm^‐1^ (the authors employed 25 mmol L^‐1^ sodium phosphate pH 7 variously supplemented with up to 1 mol L^‐1^ NaCl). Boschetti^39^ later confirmed no difference in the binding of human polyclonal antibodies to MEP HyperCel in the absence and presence of added sodium chloride, but that the addition of ammonium sulphate conveyed higher binding capacities consistent with thiophilic adsorption which is enhanced by the presence of lyotropic salts.^43^ Different behaviour is exhibited by the type III MEP‐linked adsorbent and rabbit antibodies employed here. Antiserum was diluted tenfold with 50 mmol L^‐1^ Tris–HCl pH 8 buffers supplemented with ammonium sulphate (lyotropic) or sodium chloride (chaotropic) at various concentrations up to 150 mmol L^‐1^ and contacted with adsorbents previously equilibrated in the same buffer. Figure 4(c) shows ammonium sulphate and sodium chloride data series – for percentage bound Ig and total protein and percentage purity in the adsorbed state vs conductivity – collapsing along common curves, and that increasing conductivity compromises both the level and selectivity of Ig adsorption significantly. For example, addition of 0.15 mol L^‐1^ ammonium sulphate to the clarified rabbit antiserum feedstock (which raised the electrical conductivity at 20°C from 5.6 to 31.5 mS cm^‐1^) led to >40% reduction in binding coupled with a drop in adsorbed purity from 57.5 to <45%. The salt dependent binding observed here for rabbit Ig from antiserum on MEP may in part reflect rabbit IgG's (the dominant species in the feedstock) observed tendency to dimerise as salt concentration is increased.^44^, ^45^ A reduction in Ig binding with increasing levels of salt induced dimer is consistent with the observation of reduced binding capacities for larger Ig isotypes, e.g. IgA.^39^ However, in a recent study with pure hIgG and MEP HyperCel, Yuan and coworkers^46^ noted similar binding trends at low concentrations of NaCl and (NH_4_)_2_SO_4_, i.e. steady reductions in binding capacity with increasing concentration, reaching minima at 250 mmol L^‐1^ of both salts; followed by increased binding with further addition of salt. Yuan *et al*.^46^ examined this complex behaviour using isothermal titration calorimetry, revealing that the addition of low levels of salt (0–0.25 mol L^‐1^) weakened hydrophobic interactions (causing entropy change) and strengthened van der Waals, H‐bonding and ionic interactions which led to negative enthalpy change, whereas higher concentrations (0.25–0.75 mol L^‐1^) resulted in increased hydrophobic and diminished electrostatic interactions.

### Characterisation of binding performance of type III MEP‐linked adsorbent for use in HGMF

#### Antiserum/support ratio

**Figure 5:**
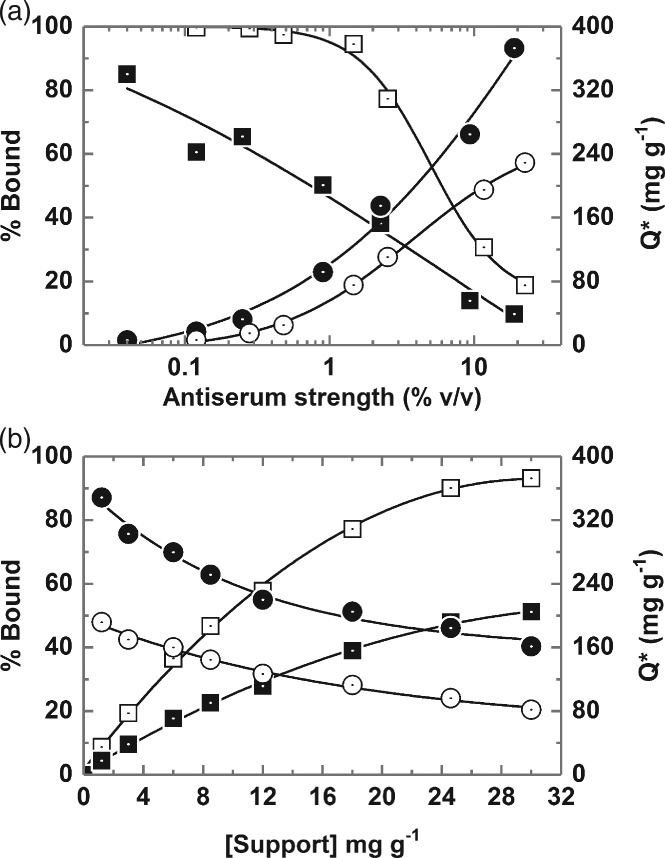
Effect of varying (a) antiserum strength (fixed support concentration = 4.6 mg mL^‐1^) and (b) support concentration (fixed 10.2% v/v antiserum feed) on Ig and total protein binding of type III MEP‐linked magnetic adsorbents. Key: percentage bound Ig (white squares); percentage bound total protein (black squares); Ig binding capacity (white circles); total protein binding capacity (black circles).

**Figure 6:**
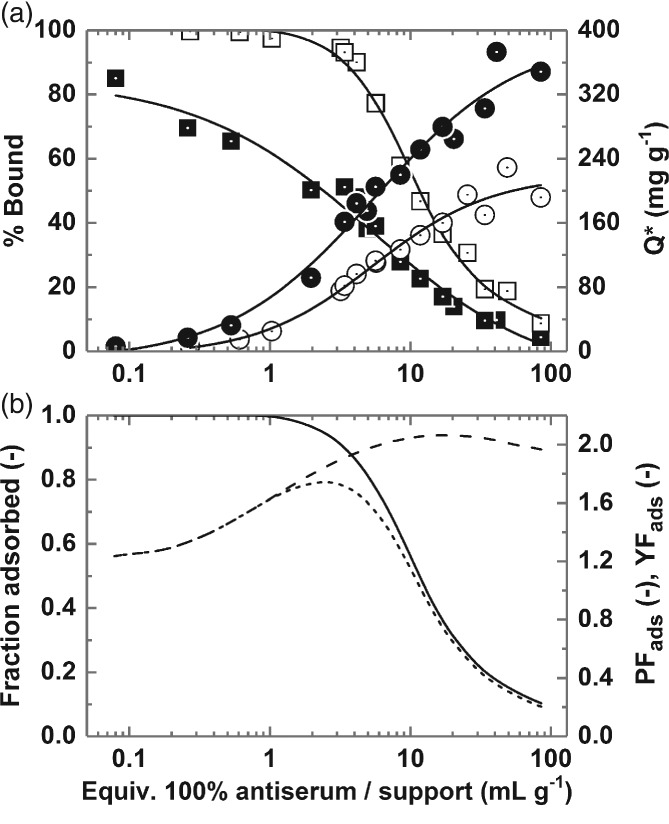
Impact of antiserum/support ratio on the binding performance of type III MEP‐linked adsorbents. Key: (a) percentage bound Ig (white squares), percentage bound total protein (black squares), Ig binding capacity (white squares), total protein binding capacity (black squares); (b) fractional Ig yield (solid line), purification factor on adsorption (PF_ads_, dashed line), yield factor on adsorption (YF_ads_, dotted line).

The most important parameter affecting the performance of any HGMF process is the magnetic adsorbent's selectivity for the target product in the feedstock from which it is to be recovered. Effective conditions for the capture of rabbit polyclonal Ig from tenfold diluted antiserum established, (i.e. adsorption time of 600 s, support equilibration and feedstock dilution with 50 mmol L^‐1^ Tris–HCl pH 8), it was subsequently necessary to further probe the equilibrium state developed within the adsorption vessel by systematically mapping the impact of antiserum/support ratio (specifically mL equivalents of 100% serum per g of support) on the immunoglobulin yield and purity achieved in the adsorption step. This was done in two ways. In the first cycle of experiments, a fixed concentration of type III MEP‐linked magnetic adsorbents 4.6 mg per mL of feedstock) was contacted with 2500–∼4.5‐fold diluted rabbit antiserum (Fig. 5(a)), representing antiserum/support ratios ranging from 0.08 – ∼49 mL equivalents of 100% antiserum per g support. In the second experimental series (Fig. 5(b)), fixed volumes of 10.2% (v/v) antiserum were mixed with various concentrations of the same support (final concentrations of 1.2–30 mg per mL of feedstock) to give a narrower, but more densely populated window of ‘antiserum/support’ challenges (3.4 to ∼85 mL equivalent to 100% antiserum per g support). The influence of antiserum/support ratio on the binding performance of type III MEP‐linked adsorbents (transposed from the data in Fig. 5(a) and (b)) is illustrated in Fig. 6. Essentially complete Ig adsorption required antiserum/support ratios <1 mL equivalent to 100% antiserum per of support (Fig. 6(a)). However, at such low antiserum/support challenges, the operational Ig binding capacity is very low (<30 mg g^‐1^) and co‐adsorption of non‐Ig proteins unacceptably high (>60% of the total protein supplied) such that the purity of the adsorbed Ig is 40% or less, corresponding with purification factors on adsorption, PF_ads_, of <1.5. The Ig binding capacity of the type III adsorbent rose strongly as the antiserum/support ratio was increased, reaching 140 and 200 mg g^‐1^ at values of 10 and 50 mL equivalent to 100% antiserum per g support, respectively, albeit at the expense of marked losses in adsorbed Ig yield (Fig. 6(a)). The total protein binding capacity of the adsorbent increased in similar manner, rising from <70 mg g^‐1^ at 1 mL equivalent to 100% antiserum per g support to 240 mg g^‐1^ at 10 mL equivalent to 100% antiserum and on past 350 mg g^‐1^ at 50 mL equivalent to 100% antiserum per g support, a value indicative from past experience with adsorbents fashioned out of the polyglutaraldehyde‐coated magnetic base particle employed here of multi‐layer binding.^23^

The degree of purification achieved on adsorption (PF_ads_) was observed to rise as antiserum/support ratio was raised, reaching a maximum value >2 at 20 mL, equivalent to 100% antiserum per g support, and thereafter declining slightly with further increase in antiserum/support ratio in keeping with the aforementioned transition from mono‐ to multi‐layer protein binding.

Identifying the correct amount of magnetic adsorbent to treat a given volume and strength of feedstock is an especially important design consideration for an HGMF process.^18^, ^19^ A favourable operating point may be defined mathematically by the yield factor, i.e. the product of the purification factor and fractional yield of the target species.^18^, ^25^ In the present case the maximum yield factor on adsorption, YF_ads_, occurs at an antiserum/support challenge of ∼3 mL equivalent to 100% antiserum per g support (Fig. 6(b)). Practically, this corresponds to treatment of a 10% (v/v) antiserum feedstock with type III support at a final particle concentration of 25–30 mg mL^‐1^. Under these conditions, >90% of the Ig present in the antiserum is adsorbed in a 1.9‐fold purified state (Purity_ads_ = 53%) at a working capacity of >80 mg g^‐1^.

**Figure 7:**
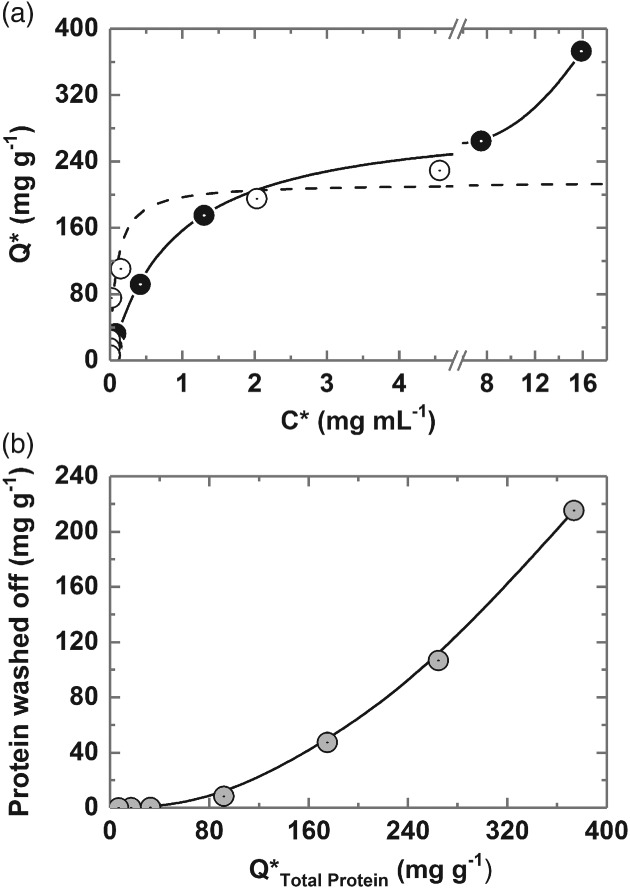
(a) Equilibrium adsorption of Ig (white circles) and total protein (black circles) from clarified rabbit antiserum on type III MEP‐linked magnetic particles. (b) Protein desorbed by washing post‐binding (grey circles) as a function of total protein binding capacity (Q*_Total Protein_). The broken line through Ig data in (a) represents the fit of the Langmuir model (Equation (1)) with the parameters Q_max_ = 213.7 ± 18.7 mg g^‐1^ and K_d_ = 0.085 ± 0.04 mg mL^‐1^.

Bak^5^ previously reported that the adsorption of rabbit antibodies to MEP HyperCel is weaker than that of human IgG and that dynamic binding capacities were roughly half those of human IgG. Despite this, and the abundance of non‐Ig proteins in rabbit antiserum esp. RSA, measurements of free Ig content in 4.5‐ to 833‐fold diluted antiserum feedstocks remaining after 0.25 h of contact confirmed strong (K_d_ < 0.1 mg mL^‐1^ ≈ 0.5 µmol L^‐1^) high capacity (Q_max_ = 214 mg g^‐1^) adsorption from the feedstock (Fig. 7(a)). Measurements of free total protein on the other hand produced a differently shaped binding curve, namely initially favourable rising towards a plateau around 200 mg g^‐1^, but then curving strongly upwards (indicative of multi‐layer binding) as the concentration of antiserum supplied is increased.^23^ The non‐selective binding nature of the MEP ligand, well documented in the case for MEP HyperCel,^6^, ^39^ is also noted for the magnetic MEP‐linked adsorbents employed herein.

Despite favourable adsorption of the target Ig, substantial non‐specific protein binding is observed at all antiserum/support challenges (Fig. 6). That this binding is weak is highlighted by the observation in Fig. 7(b) that a single rapid 30 s washing step employing the binding/dilution buffer dislodges much of the adsorbed protein resulting in substantial increases in purity of adsorbed Ig prior to elution albeit at the expense of small drops in Ig yield (see later, Tables 2 and 3). Beyond Q*_Total Protein_ ∼100 mg mL^‐1^, the amount of adsorbed protein removed by washing rises strongly as Q*_Total Protein_ is raised, reaching ∼58% at the highest loading (i.e. 215 of the 373 mg adsorbed per gram).

**Table 2:** Summary of data for magnetic rack based recovery of Ig from clarified rabbit antiserum (10% v/v) using type III MEP‐linked magnetic adsorbents. Two sets of conditions (A and B) were investigated

Condition A. Support concentration of 30 mg mL^‐1^ during binding and washing and 60 mg mL^‐1^ during elution							
Recovery step	Ig (mg)	Protein (mg)	Purity (%)	Ig yield (%)	Protein yield (%)	PF (‐)*	YF (‐)**
**Antiserum**	3.95	14.19	27.8	100	100	1.0	1.0
**Unbound**	0.27	6.93	3.9	6.8	48.8		
**Bound**	3.68	7.26	50.6	93.2	51.2	1.82	1.70
**Wash**	0.10	1.00	10.0	2.5	7.0		
**Elution 1**	1.68	2.27	73.7	42.5	16.0	2.65	
**Elution 2**	0.94	1.24	75.8	23.9	8.8	2.73	
**Combined elutions**	2.62	3.52	74.4	66.4	24.8	2.68	1.78
***Mass balance (%)***	*75.7*	*80.7*					
***Elution efficiency (%)***	*73.2*	*56.2*					‐

Condition B. Support concentration of 24.6 mg mL^‐1^ during binding and 19.5 mg mL^‐1^ during washing and elution							
Recovery step	Ig (mg)	Protein (mg)	Purity (%)	Ig yield (%)	Protein yield (%)	PF (‐)*	YF (‐)**
**Antiserum**	3.95	14.19	27.8	100	100	1.0	1.0
**Unbound**	0.39	7.38	5.3	9.9	52.0		
**Bound**	3.56	6.81	52.2	90.1	48.0	1.88	1.69
**Washes**	0.13	0.98	13.6	3.4	6.9		
**Elution 1**	2.57	3.44	74.9	65.2	24.2	2.68	
**Elution 2**	0.58	0.77	75.0	14.7	5.4	2.70	
**Combined elutions**	3.15	4.21	74.9	79.9	29.6	2.68	2.14
***Mass balance (%)***	*93.3*	*88.6*					
***Elution efficiency (%)***	*92.1*	*72.2*					

* PF = overall purification factor and

** YF = yield factor.

**Table 3:** Summary of data for HGMF based recovery of Ig from unclarified rabbit antiserum (10% v/v) using type III MEP‐linked magnetic adsorbents. The concentration of support particles employed during binding was 31.7 mg mL^‐1^ during binding and 23.5 mg mL^‐1^ during washing and elution

Recovery step	Ig (mg)	Protein (mg)	Purity (%)	Ig yield (%)	Protein yield (%)	PF (‐)*	YF (‐)**
**Antiserum**	28.7	107.6	26.67	100	100	1.0	1.0
**Flow through (unbound)**	1.35	43.52	3.10	4.7	40.4		
**Bound**	27.35	64.08	42.7	95.3	59.6	1.60	1.52
**Wash**	1.35	8.29	16.3	4.7	7.7		
**Elution 1**	15.40	19.01	81.0	53.6	17.7	3.04	
**Elution 2**	5.38	6.70	80.3	18.7	6.2	3.01	
**Combined elutions**	20.78	25.71	80.8	72.4	23.9	3.03	2.19
***Mass balance (%)***	*81.8*	*72.0*					
***Elution efficiency (%)***	*79.9*	*46.1*					

* PF = overall purification factor and

** YF = yield factor.

Attempts to reduce the level of non‐specific binding by adding sodium caprylate (an albumin‐selective moiety) to the antiserum feedstock prior to binding and/or the use of sodium caprylate wash post‐binding^39^, ^47^ were not undertaken to avoid adding ‘another layer of complexity to the purification process’.^12^ Washing with distilled water prior to elution has been reported as a means of selectively eluting non‐specifically adsorbed albumin from MEP HyperCel,^32^, ^39^ but was avoided in this work in view of Bak's^5^ observation that the water wash desorbs rabbit immunoglobulins more readily from MEP HyperCel columns than bound RSA.

### Adsorbent collection by HGMS

Before selecting conditions for elution in HGMF, it was necessary to establish the likely particle concentration under which washing and elution could be permitted by the present HGMF system. This involved defining the capacity of the magnetic filter for type III adsorbent particles from 10% (v/v) antiserum under processing conditions (support concentration = 30 mg mL^‐1^, B = 0.56 T, v = 24 m h^‐1^) and subsequently calculating the likely adsorbent concentration within the magnetic filter canister and associated recycle loop during washing and elution. 5% particle breakthrough occurred after the application of 430 mg of type III support into the magnetised filter, corresponding to a particle holding capacity of 97.7 g L^‐1^ based on the total volume of the filter. The combined volume of the recycle loop (11.6 mL) and filter cartridge (4.4 mL) minus the 430 stainless steel matrix (0.5 mL) in which desorption occurs, is 15.5 mL. Thus, assuming the support is applied at 75–90% of the magnetic filter's 5% breakthrough capacity the support concentration during desorption within the HGMF apparatus would be 20–25 mg mL^‐1^. Accordingly, a target concentration of ∼20 mg mL^‐1^ of protein loaded adsorbents was selected for optimisation of elution conditions.

### Optimisation of elution conditions for HGMF

**Figure 8:**
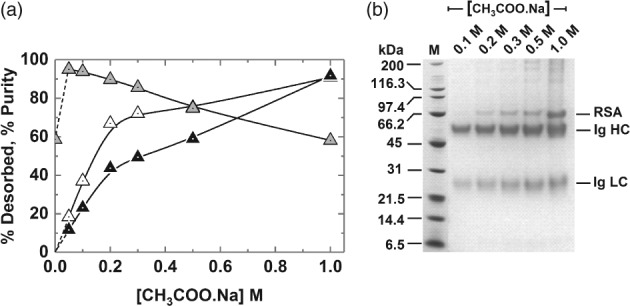
Effect of sodium acetate pH 4 buffer concentration on the (a) desorption of bound rabbit Ig and total protein from washed type III MEP‐linked magnetic adsorbents in a single elution cycle and (b) corresponding SDS‐PAGE analysis. The support concentration during binding was 24.6 mg mL^‐1^ and was reduced to 19.5 mg mL^‐1^ for washing and elution. The amounts of Ig and total protein released are expressed as percentages of the total bound prior to elution. Key: desorbed Ig (white up‐triangles), desorbed protein (black up‐triangles); Ig purity (grey up‐triangles).

In preliminary tests, varying the pH of the 50 mmol L^‐1^ acetate buffer, by ± 0.5 units from the initial value of 4 employed in screening the different MEP‐linked supports, gave no improvement in desorption efficiency from washed protein‐laden supports, and neither did the use of 100 mmol L^‐1^ sodium citrate buffers pH 3 and 3.5. We therefore evaluated the strength of acetate buffer needed to confer the necessary driving force for Ig release (Fig. 8). For this, type III magnetic MEP‐linked adsorbent particles were contacted with 10% (v/v) antiserum at a final support concentration of 24.6 mg mL^‐1^ in tenfold diluted serum, washed once with binding buffer at a support concentration of 19.5 mg mL^‐1^ and then mixed at the same concentration with 50 mmol L^‐1^–1 mol L^‐1^ sodium acetate buffers, pH 4. Immediately post‐binding the purity of adsorbed Ig registered 52%. Following washing, the adsorbed purity increased to nearly 60% at the expense of a 10% loss in yield (indicated in Fig. 8(a) by data point at 0 mol L^‐1^ sodium acetate). At the lowest sodium acetate concentrations of 0.05 and 0.1 mol L^‐1^ the first elution step selectivity of immunoglobulin elution was high (the purity of desorbed Ig was >93% by assay), however the recoveries were poor (<20% and < 40% for the 0.05 and 0.1 mol L^‐1^ sodium acetate elution buffers, respectively). RSA, the major impurity, is barely visible on reducing SDS‐polyacrylamide gels, representing just 2% of the combined Ig heavy and light chain intensities (Fig. 8(b)). Desorption yield increases as the elution buffer strength is raised, but comes at the expense of impaired selectivity, emphasised by a roughly linear decline in purity by assay (Fig. 8(a)) and corresponding growth in RSA contamination (reaching >25% of the Ig signal at 1 mol L^‐1^ sodium acetate) and of higher molecular weight species (Fig. 8(b)). Although a single elution step employing 1 mol L^‐1^ sodium acetate desorbs 91% of the initially adsorbed Ig, all selectivity is lost (Fig. 8(a), compare percentage purities at 0 and 1 mol L^‐1^ sodium acetate). A further issue encountered with elution using 1 mol L^‐1^ sodium acetate elution buffer, but not the lower concentrations employed, was the formation of a clear precipitate (most likely of lipoprotein^4^, ^5^, ^6^) after freezing and thawing the eluates. For these reasons, a sodium acetate buffer concentration of 0.5 mol L^‐1^ was selected for use in subsequent HGMF and small‐scale purification tests, as the best compromise of yield and purity.

### Recovery of Ig from rabbit antiserum feedstocks

#### Magnetic rack based Ig purification from clarified feed

**Figure 9:**
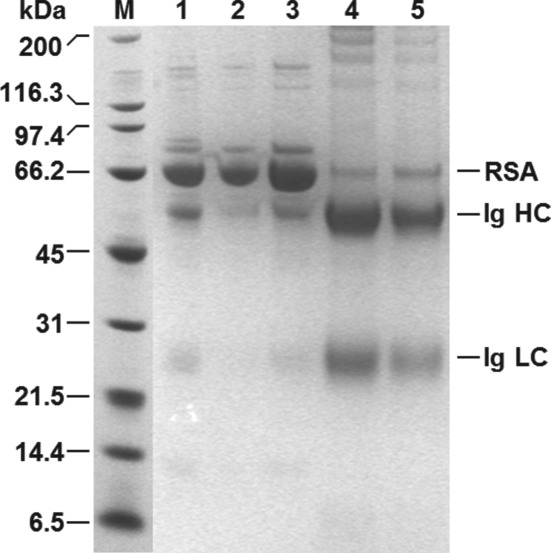
Reducing SDS‐PAGE analysis of ‘Table 2 Condition B' samples arising from magnetic rack based Ig recovery from clarified 10% (v/v) rabbit antiserum using type III MEP‐linked magnetic supports. Key: molecular weight markers (M); rabbit antiserum (1); unbound (2); wash (3); elution 1 (4); elution 2 (5); RSA = rabbit serum albumin; Ig = immunoglobulin; HC = heavy chain; LC = light chain.

Before conducting HGMF at larger scale, small magnetic rack based purifications were performed to further examine the effects of support concentration during washing and elution on Ig purification performance. Two sets of conditions were employed, i.e. condition ‘A' – binding and washing at support concentration of 30 mg mL^‐1^ and elution at 60 mg mL^‐1^; and condition ‘B' – binding at a support concentration of 24.6 mg mL^‐1^ and washing and elution at 19.5 mg mL^‐1^. Table 2 summarises the data obtained and Fig. 9 shows the electrophoretic analysis corresponding to condition B.

In keeping with earlier findings (Figs 5 and 6), the higher support concentration during condition A's binding step resulted in higher Ig binding yield (93.2% of that initially present in the antiserum), but lower purity in the adsorbed state (50.6%) cf. condition B (90.1% bound, Purity_ads_ = 52.2%). The use of a higher support concentration (condition A) during washing also improved the selectivity of this step. While very similar amounts of total protein were desorbed in both conditions, the amount of bound Ig lost from the supports was >30% lower for condition A (Table 2). Scrutiny of the electrophoretogram (Fig. 9, lane 3) for condition B confirms that most of the protein washed from the supports was RSA; only a faint trace of Ig is visible. Immediately prior to elution, the Ig yield and purity combinations were little different for the two conditions, emphasised by the similarity in purification and yield factors at this stage of ∼1.85 and ∼2.1 for conditions A and B, respectively (Table 2).

The biggest impact on purification performance was the support concentration used during elution. For condition B (19.5 mg mL^‐1^) two successive elution cycles recovered >92% of the Ig remaining bound on supports after washing, leading to an overall Ig recovery of ∼80%. Calculated desorption efficiencies for the first and second cycles were similarly high (i.e. >75% in the first, dropping to >68% in the second). Desorption efficiency was markedly reduced when a more than threefold higher support concentration (60 mg mL^‐1^) was employed (condition A), falling to 46.9 and 49.7% in the first and second cycles, respectively. As a result the overall yield registered 66.4%, but the purity was not affected (Table 2).

#### HGMF based Ig purification from unclarified feed

**Figure 10:**
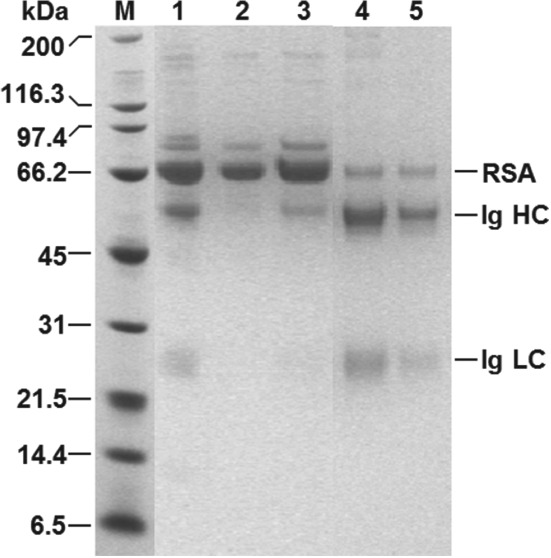
Reducing SDS‐PAGE analysis of ‘Table 3’ samples arising from HGMF based Ig recovery from unclarified 10% (v/v) rabbit antiserum using type III MEP‐linked magnetic supports. Key: molecular weight markers (M); rabbit antiserum (1); flow through (2); wash (3); elution 1 (4); elution 2 (5); RSA = rabbit serum albumin; Ig = immunoglobulin; HC = heavy chain; LC = light chain.

The limited availability of feedstock constrained the scale at which HGMF could be demonstrated and the type of device that could be used, and permitted only a single run to be conducted. The results from small‐scale magnetic rack studies (Table 2) were thus crucial in informing selection of conditions for HGMF from unclarified 10% (v/v) antiserum. The support concentration during binding was increased to 31.7 mg mL^‐1^ (cf. 24.6 and 30 mg mL^‐1^ for conditions B and A, respectively; Table 2), and the adsorbent concentration during the wash and elution steps was 23.5 mg mL^‐1^. The same binding and elution times were employed (i.e. 600 s in both cases), but given the presence of solids in the unclarified antiserum feed and to ensure efficient particle release from the magnetic filter, the duration of the single wash cycle was raised from 30 to 60 s. Table 3 summarises the results obtained and Fig. 10 displays the corresponding SDS‐PAGE analysis of fractions from the run.

In general, it is clear that purification transferred successfully from magnetic rack (Table 2) to HGMF operation (Table 3) and that the added presence of suspended solids in the unclarified antiserum did not perturb overall Ig purification performance, as the combined yield of >72% from two elution cycles was similar and the calculated Ig purity of 81% was slightly higher than that achieved in smaller scale tests with the clarified antiserum feedstock. Closer scrutiny of the data obtained at each sub‐step however, highlights differences arising from the feedstock (i.e. unclarified vs clarified) and/or magnetic particle separation mode (i.e. magnetic filter vs magnetic rack) employed.

The slightly higher support concentration employed during binding resulted in capture of >95% of the Ig from the unclarified antiserum (Table 3); but this small gain was offset by a larger increase in the amount of protein adsorbed onto the supports (i.e. to nearly 60% cf. 51–52%), thus the calculated purity in adsorbed state prior to washing was just 43% cf. 51–52% observed in the smaller scale magnetic rack experiments conducted with the clarified feed (Table 2). After washing, the amount of protein adsorbed on the support dropped significantly (to 52%), but not to the level expected from smaller scale magnetic rack conducted with clarified antiserum (i.e. 41–44%), while the amount of adsorbed Ig lost into the wash increased to 4.7% (Table 3). Both effects probably stem from ‘additional’ surface fouling from extra components in suspended solids in unclarified feed. The selectivity of Ig desorption from supports during elution in HGMF was not affected by the increased level of competing species adsorbed on the non‐porous surfaces of the magnetic MEP‐linked adsorbent particles (compare entries for percentage eluted of firmly bound, purity and purification factor in Table 3 with those in Table 2).

The banding patterns observed in the electrophoretogram of HGMF fractions (Fig. 10) are qualitatively very similar to those from the small‐scale condition B (Fig. 9) and are entirely consistent with the process data in Table 3. Bands for Ig cannot be detected in lane 2 (flow through) and the intensity of the RSA band is much reduced cf. lane 1 (starting material). A strong RSA band intensity is restored in lane 3 (wash) accompanied by faint heavy and light chain Ig bands. The relative intensities of RSA and Ig bands are reversed in lanes 4 and 5, respectively, corresponding with the first and second cycles of elution.

Calculated Ig desorption efficiencies (59.2% for the first elution dropping to 50.5% for the second) were lower than expected from small‐scale studies conducted with the ‘cleaner’ clarified feed (see above). Nearly 80% of the firmly bound Ig was retrieved by these two HGMF elution cycles (Table 3), but attempts to recover ‘missing’ Ig remaining bound on supports proved unsatisfactory. While four additional cycles dislodged a further 2.15 mg of Ig (8.2% of that firmly bound before elution), boosting the overall Ig yield from 72.4% to 79.9%, Ig purity fell to 60.2% because an extra 12.3 mg of protein (22% of that firmly bound before elution) was desorbed from the supports. After the last elution cycle had been completed, the ‘charge’ of magnetic particles within the HGMF system was flushed out and the filter was dismantled for cleaning. Visual inspection of the unrolled filter matrix confirmed the trace presence of magnetic particles, but no biological fouling material was evident.

#### Limitations of HGMF system employed

The main advantage of the mini‐pilot‐scale cyclically operated ‘ON–OFF’ permanent magnet based HGMS used in this work is that it allows demonstration of the HGMF concept with small volumes of feedstock. Despite the inherent novelty of its design, it does not reflect the current ‘state‐of‐the‐art’. In common with earlier studies^19^, ^20^, ^22^, ^23^, ^24^, ^25^, ^26^, ^41^, ^42^ the system used in this work employs a canister packed with a matrix of ferromagnetic wires and an extracorporeal recycle loop for conducting washing and elution cycles when the field is switched ‘OFF’. While this design is capable of delivering high particle separation efficiencies from complex feed streams and affords powerful demonstrations of the processing speed and capabilities of HGMF for recovering candidate protein targets from highly complex unclarified bioprocess feedstocks, as illustrated in this study, it does not lend itself to multi‐cycle operation^26^ and potential exploitation at large scale.^15^, ^21^

Two weaknesses of the ‘fixed filter matrix + recycle loop’ device are apparent from the work conducted here. First, the much higher product and support concentrations employed in this work cf. previous studies,^18^, ^20^, ^23^, ^24^, ^25^, ^26^, ^41^, ^42^ rendered concentration on elution impossible. Loading of the ‘feedstock + particle’ cocktail into the filter took just 25 s. Although particles were concentrated nearly threefold within the filter, following two steps of elution, which recovered 80% of the adsorbed Ig, the combined eluate's Ig concentration was 3.7‐fold more dilute than that of the feed (i.e. 0.67 cf. 2.5 mg mL^‐1^). Second, in the ‘fixed filter matrix + recycle loop’ design, adsorbents are flushed out of the filter matrix and mixed with eluant by recirculating the resulting suspension at high velocity around the canister–loop circuit. For product elution from the adsorbent particles to be effective, efficient particle release from matrix wires must occur. The reduced elution efficiency identified above, combined with detection of adsorbent particles within the filter at the end of the experiment, point to incomplete particle release.

#### Rotor–stator HGMS

The aforementioned problems, additional related issues and requirements for automated multicycle operation and cGMP compliance have driven the development of the automated rotors–stator magnetic filters.^9^, ^15^, ^21^, ^27^ Rotor–stator HGMS devices feature two sets of alternating perforated stainless steel filter discs – one set can be rotated at high speed and the other is stationary. The design is highly effective at re‐slurrying attached magnetic particles at zero field, affords high particle collection capacities (>200 g per litre of filter) and continuous multi‐cycle operation without loss in performance from one operating cycle to the next. All washing and elution steps are conducted within the separator, eliminating the need for extracorporeal recycle loops or external vessels, leading to reduced buffer consumption, simpler and faster operation. Against the above, it is reasonable to conclude that had a modern rotor–stator HGMS been employed to recover and process product laden type III MEP‐linked magnetic adsorbents from the same feedstock, all aspects of Ig purification performance by HGMF would have markedly improved.

## CONCLUSIONS

An HGMF process for the recovery of rabbit Ig from unclarified antiserum using MEP‐linked non‐porous superparamagnetic adsorbents has been developed. Unconditioned ultra‐high titre bioprocess liquors present stern challenges to adsorptive separation techniques, and though specifically designed for capture from crude complex feeds, HGMF is not immune to these. To recover >90% of the Ig present in raw unclarified serum (25 g Ig per L; 93 g protein per L) would require ∼0.3 g type III MEP‐ linked adsorbent per mL, which is roughly 3‐fold higher than both the adsorbent holding capacity of the magnetic filter used in this work and the support's sediment density.^48^ Because of this antiserum was diluted tenfold bringing the target Ig titre to 2.5 g L^‐1^, a level similar to that of monoclonal antibody‐containing culture broths.^12^ Greater than 95% of the Ig present in this unclarified feed was adsorbed at a working Ig binding capacity of 75 mg g^‐1^, and after a brief washing step 80% of the bound Ig was recovered in two elution cycles in more than threefold purified form (81% uncorrected; 90% corrected) appropriate for immunodiagnostic use. The whole process took 0.5 h, but considerable time savings (principally by reducing the binding time) are clearly possible without sacrificing purification performance.

To cope with higher Ig titres found in animal sera, human plasma and highly expressing CHO cultures, more selective adsorption is required to make full use of the available surface area for target binding (200–250 mg g^‐1^ with present magnetic support design) thereby reducing the amounts of magnetic support required to treat a given feed. This could be achieved either through the prior and/or post addition of sodium caprylate^39^, ^47^ to reduce serum albumin adsorption (the potential downsides here are reduced Ig yield and compromised isolation of other targets), boosting the immobilised ligand density – perhaps through grafting of ligand‐bearing polymers or dendrons,^15^, ^49^ and/or use of more potent ‘capture’ ligands.^12^, ^36^
